# High expression of CXCR4 may predict poor survival in resected pancreatic adenocarcinoma

**DOI:** 10.1038/sj.bjc.6605020

**Published:** 2009-04-07

**Authors:** R Maréchal, P Demetter, N Nagy, A Berton, C Decaestecker, M Polus, J Closset, J Devière, I Salmon, J-L Van Laethem

**Affiliations:** 1 Department of Gastroenterology, GI cancer Unit, Erasme University Hospital, Université Libre de Bruxelles, Brussels, Belgium; 2 Department of Pathology, Erasme University Hospital, Université Libre de Bruxelles, Brussels, Belgium; 3 Laboratory of toxicology, Institute of Pharmacy, Université Libre de Bruxelles, Brussels, Belgium; 4 Departments of Medical Oncology, Centre Hospitalier Universitaire Sart -Tilman, Liège, Belgium; 5 Department of Digestive Surgery, Erasme University Hospital, Université Libre de Bruxelles, Brussels, Belgium

**Keywords:** pancreatic adenocarcinoma, chemokine receptor, metastases, hypoxia inducible factor-1

## Abstract

Chemokines and their receptors are involved in tumourigenicity and clinicopathological significance of chemokines receptor expression in pancreatic adenocarcinoma (PA) is not fully understood. This study was conducted to determine patients' outcome according to the expressions of CXCR4, CXCR7 and HIF-1*α* after resection of PA. Immunohistochemistry for CXCR4, CXCR7 and HIF-1*α* expressions as well as cell proliferative index (Ki-67) was conducted in 71 resected (R0) PA and their 48 related lymph nodes (LN) using tissue microarray. CXCR4 and CXCR7 expressions were positively correlated to HIF-1*α* suggesting a potential role of HIF-1*α* in CXCR4 and CXCR7 transcription activation. Patients with CXCR4^high^ tumour expression had shorter OS than those with low expression (median survival: 9.7 *vs* 43.2 months, *P*=0.0006), a higher risk of LN metastases and liver recurrence. In multivariate analysis, high CXCR4 expression, LN metastases and poorly differentiated tumour are independent negative prognosis factors. In a combining analysis, patients with CXCR4^low^/CXCR7^low^ tumour had a significantly shorter DFS and OS than patients with a CXCR7^high^/CXCR4^high^ tumour. CXCR4 in resected PA may represent a valuable prognostic factor as well as an attractive target for therapeutic purpose.

Pancreatic adenocarcinoma (PA) is characterised by early locoregional spread and distant metastasis. As a result, most of the patients are unresectable at the time of diagnosis. Without treatment, life expectancy of such patients is only 3–6 months (Burris *et al*, 1997; Jemal *et al*, 2007). Despite curative surgery the 5-year overall survival (OS) is around 20%. New strategies and therapies are urgently needed whereas we also need to identify new prognostic and predictive biomarkers. A lot of different mechanisms, mediators and pathways have been investigated trying to explain the PA aggressive phenotype (Furukawa *et al*, 2006). Chemokines and their receptors are implicated in the development of different types of cancers (Müller *et al*, 2001; Schimanski *et al*, 2005; Nakamura *et al*, 2006; Yasumoto *et al*, 2006). In breast and gastric adenocarcinoma, tumour growth, angiogenesis as well as the homing of tumour cells to the sentinel lymph nodes and distant predilection sites are mediated by chemokines and their receptors (Müller *et al*, 2001; Schimanski *et al*, 2005; Nakamura *et al*, 2006; Yasumoto *et al*, 2006). Chemokines act through specific 7-transmembrane receptors coupled to G proteins (GPCR). They are present on almost all cell types but were initially identified on leucocytes, where they are known to play a major role in the inflammatory process (Proudfoot, 2002).

Stromal-derived factor-1*α* (SDF-1*α* or CXCL12) is a broadly expressed CXC chemokine which serves as a potent chemoattractant for mature and immature haematopoietic cells. High levels of CXCL12 are produced in lymph nodes, liver, lung, bone marrow or brain. They are common sites of pancreatic metastases suggesting that this chemokine accounts for the homing of pancreatic cancer cells to specific organs. The predominant CXCL12 receptor is the CX chemokine receptor 4 (CXCR4), a protein frequently overexpressed on the surface of human tumour cells of epithelial origin (Scotton *et al*, 2002; Cunha *et al*, 2003; Hartmann *et al*, 2005; Kaifi *et al*, 2005; Kim *et al*, 2005; Schimanski *et al*, 2005). Most pancreatic cancer cell lines also express CXCR4. In CXCR4-positive PA cell lines, CXCL12 not only enhances chemotaxis, transendothelial migration and matrigel invasion, but also stimulates cell proliferation and protect them from serum deprivation-induced apoptosis (Koshiba *et al*, 2000; Marchesi *et al*, 2004; Saur *et al*, 2005).

CXCL12 can bind another chemokine receptor, such as CXCR7, which is present on the surface of many different malignant cell types (Burns *et al*, 2006), on tumour-associated blood vessels, but not on normal vasculature (Miao *et al*, 2007). CXCR7 promoted the survival of tumour cells by preventing apoptosis, increased adhesion properties and dissemination, but did not mediate chemotaxis towards CXCL12 (Burns *et al*, 2006). CXCR7 induces proliferation of lung, prostatic and breast cancer cell lines (Burns *et al*, 2006; Miao *et al*, 2007; Wang *et al*, 2008) and tumour growth enhancement and dissemination in a breast cancer xenograft mouse model (Burns *et al*, 2006; Miao *et al*, 2007). Although its role in pancreatic cancer development still needs to be determined, CXCR7 has been supposed to play a role in the pathogenesis of pancreatic cancer.

The hypoxia inducible factor-1 (HIF-1) induces upregulation of CXCR4 transcription and CXCR4 transcripts stabilisation (Staller *et al*, 2003; Li *et al*, 2004). For this reason, CXCR4 was proposed to be responsible for enhanced malignancy of tumour cells in hypoxic areas (Schioppa *et al* 2003; Staller *et al* 2003; Schutyser *et al*, 2007).

In our study, we evaluated CXCR4, CXCR7 and HIF-1*α* expressions, their relative impact in the outcome of resected PA patients and whether these three biomarkers are correlated with clinicopathological factors.

## Materials and methods

### Patients and tissues

Patients with primary PA who had undergone curative surgery were retrospectively reviewed. Seventy-eight specimens of Whipple resection and partial pancreatectomy during the period of 1998–2006 for PC were identified and their formalin-fixed paraffin-embedded (FFPE) tissue blocks were retrieved in our Department of Pathology. A complete microscopic resection (R0), including circumferential retroperitoneal margin evaluation, was achieved for 71 out of the 78 and they constituted our study population.

### Tissue microarray construction

Tissue cores were obtained from FFPE and slides of each case have been reviewed by a pathologist (PD). Based on the haematoxylin–eosin staining, representative areas of the tumour (tumour bulk and invasive margin) and the lymph node (LN) metastasis have been selected. Five tissue cores of 0.6-mm diameter in each of the three areas mentioned above were placed into a recipient block by using a precision arraying instrument (Beecher Instruments; Micro Tissue Arrayer, Silver Springs, MD, USA). We constructed four TMA blocks containing a total of 950 cores of ductal adenocarcinoma. Five-micrometer sections were cut from completed array blocks and transferred to adhesive slides. Slides were protected against antigen deterioration by paraffin coating before their use. Sections were stained with haematoxylin and assessed for adequate tumour representation.

### Immunohistochemistry

Immunohistochemical staining of FFPE tumour tissue was performed using non-biotinylated rabbit polyclonal anti-human antibodies against CXCR7 (Abcam, Cambridge, UK; dilution 1 : 100), mouse monoclonal anti-human CXCR4 (R&D Systems Inc., Minneapolis, MN, USA; dilution 1 : 200), mouse monoclonal anti-human HIF-1*α* (R&D Systems Inc.; dilution 1 : 20) and mouse monoclonal anti-human Ki-67 (Dako, Heverlee, Belgium; clone Mib-1, dilution 1 : 1000). Antigen retrieval was performed by microwave pretreatment in 0.01 M citrate buffer for 10 min (CXCR7, Ki-67). Sections were incubated with mouse anti-rabbit or goat anti-mouse secondary IgG biotinylated secondary antibody for 30 min. Immunoreactivity was visualised by means of avidin–biotin–peroxydase complex kit reagents (Biogenex, San Ramon, CA, USA) as the chromogenic substrate. Finally, sections were weakly counterstained with Mayers haematoxylin, and mounted. Hodgkin's lymphoma (CXCR4), kidney (CXCR7), lymph node (Ki-67) and prostatic adenocarcinoma (HIF-1*α*) were used as positive controls. Irrelevant rabbit or mouse IgG antibodies were applied to negative control.

### Immunostaining grading and score

We used the Spot Browser V2e (Alphélys, Plaisir, France) software controlling a camera connected to a microscope for the automatical acquisition of each spot. Slides were scanned using a customised computer-controlled microscope (Olympus BX50 (Olympus, Aartselaar, Belgium) with *xy*-stage and *z* controller). Images of each spot are acquired automatically with a high-resolution camera (DXC-390; Sony, Londerzeel, Belgium). Spots were examined independently by two observers (PD and RM) blinded to both clinical and pathological data. Immunoposivity was assessed with respect to cellular localisation (membranous, cytoplasmic or nuclear), intensity and distribution. Proliferative activity was assessed by scoring the percentage of labelled cells. Expressions of CXCR7, CXCR4 and HIF-1*α* were quantified using a visual grading system based on the extent of staining (percentage of positive tumour cells graded on scale from 0 to 3: 0, none; 1, 1–30%; 2, 31–60%; 3, >60%) and the intensity of staining (graded on a scale of 0–3: 0, none; 1, weak staining; 2, moderate staining; 3, strong staining). The combination of extent (E) and intensity (I) of staining was obtained by the product of E × I called EI varying from 0 to 9 for each spot. The mean EI score was calculated for each PA specimen (tumour bulk + invasive margin) and their related LN metastasis.

For statistical analysis, EI score of 0–3 were considered low expression and EI score >3 were considered high expression. This cut-off value was based on the immunostaining pattern observed for CXCR4, HIF-1*α* and CXCR7 expressions (see below). In case of CXCR4, CXCR7 and HIF-1*α* expressions, staining intensity was quite homogeneous within tumour specimens and all epithelial cells of adenocarcinoma express CXCR4, CXCR7 and HIF-1*α*. Therefore, specimens differ essentially in terms of staining intensity.

### Validation of the microarray cohort

Spots were excluded from the analysis if they were no tumour or minimal tumour specimen. We decided that at least two valuable spots per area of interest are required to calculate the mean EI score per area. Of the 71 patients, 70 (97.2%) were assessable for each of the tumour areas located in the primary tumour. Of 48 patients with LN metastasis, 37 (70%) were assessable for LN tumour cells protein expression.

### Statistical analysis

The primary outcome variables were OS and DFS. Survival curves were estimated using the Kaplan–Meier method (Kaplan and Meier, 1958) and differences among groups were analysed using the log-rank test. OS was calculated as the period from the day of surgical resection to death of any cause or until the date of the last follow-up, at which point data were censored. DFS was defined as the time between surgical resection until either of progression of disease, death from any cause and last radiological assessment. Data on survivors were censored at the last follow-up. We used non-parametric tests to compare independent groups of numerical data (Mann–Whitney test) and categorical data (*χ*^2^-test and Fisher's exact test). The correlations among numerical variables were assessed by Spearman's rank correlation analysis. Multivariate analyses, with backward variable selection, were conducted using the Cox's proportional-hazards regression model (Cox, 1972). Variables at the 0.10 level in univariate analysis were included in the multivariate model. The level of significance was defined as *P*<0.05. All the statistical analyses were carried out using the Statistical Package for Social Science 11.0 software (SPSS Inc., Chicago, IL, USA).

## Results

### Patients 'characteristics

Seventy-one patients constituted our case series. Characteristics of the patients are listed in Table 1. There were 39 men and 32 women with a median age of 64.5 years (range: 39–81 years). All patients underwent a R0 duodenopancreatectomy and none were treated with neoadjuvant therapy. Five patients (7.1%) died from surgical complications within 1.5 months after resection. These patients were excluded from survival analysis. Adjuvant treatment was administered in 42 patients (59%) and consisted in a combination of chemoradiation for 30 of them and gemcitabine monotherapy for 12. The median OS was 16.4 months (range: 3.2–107.4) and the median DFS of 10.1 months (range: 1.4–107.4). Of the 71 patients, 49 (69%) relapsed (11 locally, 25 in distant organs, 13 both locally and in distant organs) and 38 received chemotherapy after disease relapsing. At the end of the follow-up period, 17 patients (24%) were free of disease recurrence.

### Expression pattern of CXCR4, CXCR7 and HIF-1*α* in PA

CXCR4 was expressed in 60 out of 71 tumours (84.5%; Table 2). Of the 71 tissues, 65 had uniformly detectable CXCR4 immunostaining; 6 samples displayed a proportion (<30%) of adenocarcinoma cells without undetectable CXCR4. CXCR4 staining showed a predominantly cytoplasmic distribution in the cancer cells.

CXCR7 expression was restricted to the cytoplasm and present in 53 out of 71 (74.6%) tumours (Table 2). Like CXCR4, the expression was homogeneous within a determined specimen. No staining was observed in the normal acinar and ductal cells of the peritumoural areas. Weak staining for CXCR4 and CXCR7 was observed in a majority of the infiltrating inflammatory cells. Furthermore, CXCR4 and CXCR7 were highly expressed in tumoural blood vessels whereas no staining was observed in the endothelium of blood vessels in normal pancreatic tissue.

**Figure 1:**
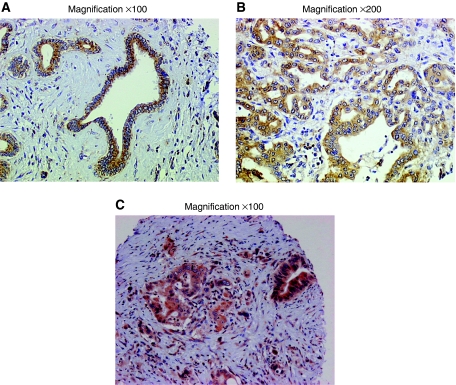
Immunostaining for CXCR7, HIF1-*α*, CXCR4 in pancreatic adenocarcinoma. Pancreatic adenocarcinoma showing strong cytoplasmic staining for CXCR7 (**A**), HIF1-*α* (**B**), CXCR4 (**C**). Adjacent lymphocytes (**A**) and (**C**) demonstrate staining and provide a positive control. (**A**) Magnification × 100; (**B**) Magnification × 200; (**C**) Magnification × 100.

HIF-1*α* was predominantly identified into the cytoplasm of cancer cells in 48 out of 71 (67.6%) tumours; and 11 out of 71 (15.5%) displayed nuclear staining (Figure 1). Table 2 summarises CXCR4, CXCR7 and HIF-1*α* expressions in the tumours and in their related LN metastasis.

### Correlation among CXCR4, CXCR7 and HIF-1*α* expressions

HIF-1*α* expression was positively correlated with those of CXCR4 and CXCR7 among tumour specimens (HIF-1*α*-CXCR4, Spearman's *r*=0.55, *P*<0.0001; HIF-1*α*-CXCR7, Spearman's *r*=0.29, *P*=0.013).

### Correlation among CXCR4, CXCR7, HIF-1*α* expressions and clinicopathological factors

LN metastasis (*P*=0.005) and liver recurrence (*P*=0.016) were significantly higher in PA showing high CXCR4 expression than in those with low expression. None of the other parameters − cancer stage, tumour differentiation, vascular and perinervous embols − had a significant relationship to CXCR4 expression (Table 3). We did not found any association among HIF-1*α*, CXCR7 tumour expression and the different clinicopathological factors analysed in this study (data not shown).

### Relationship between CXCR4/CXCR7 expression and proliferative index

**Figure 2:**
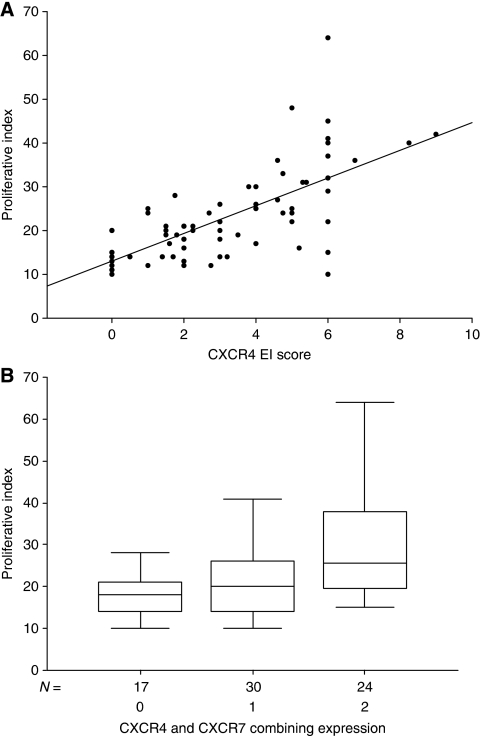
(**A**) Increasing proliferative index correlate significantly with rising EI scores for CXCR4 expression in pancreatic cancer (*P*<0.001). (**B**) Proliferative index according to CXCR4 and CXCR7 expressions: 0=CXCR4^low^/CXCR7^low^, 1=CXCR4^high^/CXCR7^Low^, 2=CXCR4^high^/CXCR7^high^.

The mean proliferative index (PI) for pancreatic tumours was 22.97±10.37%. There was a significant positive linear correlation between PI and CXCR4 EI score (Spearman's *r*=0.583, *P*<0.001; Figure 2A), but not between CXCR7 EI score and PI. Interestingly, PI was significantly higher in CXCR4^high^/CXCR7^high^ tumours than in CXCR4^low^CXCR7^low^ tumours (mean PI: 29.08±12.33 *vs* 18.06±5.01, *P*=0.001; Figure 2B).

### Association of high CXCR4 expression with poor survival outcome

**Figure 3:**
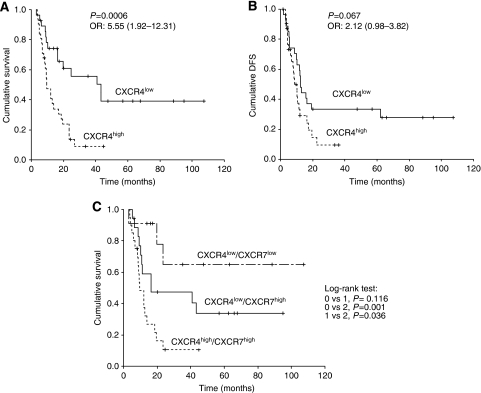
Kaplan–Meier analysis of survival (**A**) and DFS (**B**) according to CXCR4 expression. (**C**) OS according to subgroup combining analysis.

In the univariate analysis, LN metastasis, poorly differentiated tumour and CXCR4 expression predicted poor survival and shorter DFS (Table 4). Patients with high CXCR4 tumour expression clearly had a worse outcome than those with low CXCR4 expression (OS: 9.7 months (95% CI: 6.0–13.4) *vs* 43.2 months (95% CI: 16.3–78.1), *P*=0.0006; DFS: 8.6 months (95% CI: 5.8–11.3) *vs* 12.4 months (95% CI: 10.7–14.0, *P*=0.067; Figure 3). Patients with LN metastasis (OS: 12.6 (95% CI: 5.8–19.3) *vs* 43.3 months (95% CI, 3.3–75.8), *P*=0.008; DFS 10.1 (95% CI: 8.0–12.1) *vs* 22.6 months (95% CI: 4.0–83.9), *P*=0.003) and poorly differentiated tumour (OS: 18.6 (95% CI: 8.3–28.9) *vs* 8.8 months (95% CI: 5.4–12.2), *P*=0.03 and DFS: 12.1 (95% CI: 6.0–18.7) *vs* 6.3 months (95% CI: 3.0–9.5), *P*=0.002) were also associated with short DFS and OS.

In multivariate analysis (Table 5), CXCR4 expression, poorly differentiated tumour and LN metastasis were negative independent prognostic factors for OS whereas LN metastasis and poorly differentiated tumour were negative prognostic indicators for DFS.

### CXCR4 and CXCR7 coexpression and patients outcome

Interestingly, patients with CXCR4^high^/CXCR7^high^ tumour expression have a significantly prolonged DFS and OS than those patients with a CXCR4^low^/CXCR7^low^ tumour expression (Figure 3C; median DFS: 15.80 months (95% CI: 10.43–21.17) *vs* 7.62 (2.97–12.27), *P*=0.037; median OS: not reached *vs* 9.69 (95% CI: 5.13–14.07), *P*=0.001).

## Discussion

In this study, we used immunohistochemical methods applied to TMA technology to evaluate CXCR4, CXCR7 and HIF-1*α* expressions in PA tissues and their impact on patients' outcome. The CXCR4 staining was predominantly cytoplasmic, whereas a few cases presented an additional nuclear and/or membranous localisation. A similar pattern has been recently described (Maeda *et al*, 2008) and is explained by the translocation of CXCR4 from the membrane to the cytoplasm (Burger *et al*, 2003). CXCR4, CXCR7 and HIF-1*α* proteins expressions were homogeneous within the different intratumour areas and no difference of expression was detected between the primary and LN metastasis. Positive correlations among the CXCR4, CXCR7 and HIF-1*α* expressions were found suggesting a possible role of HIF-1*α* in the transcription activation of CXCR4 and CXCR7. Hypoxia and HIF-1*α* is known to induce the transcription of CXCR4 (Staller *et al*, 2003) and other chemoreceptors including CCR7, CXCR1 and CXCR2 (Wilson *et al*, 2006; Maxwell *et al*, 2007). It is credible that CXCR7 can be one of the multiple genes targeted by HIF-1*α* suggesting that hypoxia can trigger chemokines expression and enhance malignancy of tumour cells in hypoxic area.

Data from *in vitro* and murine *in vivo* tumour models underline the critical role of CXCR4/CXCL12 receptor ligand system for pancreatic tumour cells. The CXCR4/CXCL12 system operates probably as a paracrine loop (Mori *et al*, 2004) to enhance the malignancy of pancreatic cancer cells. This enhanced malignancy was generally attributed to a role of CXCR4 in cells migration, matrix degradation and tissue invasion, as the ligand CXCL12 is present in many tissues and thought to promote migration into these tissues (Koshiba *et al*, 2000; Marchesi *et al*, 2004; Mori *et al*, 2004; Saur *et al*, 2005). Potential relationship among CXCR4, CXCR7 expressions and cell proliferation was explored. There was a significant positive correlation between the EI score of CXCR4 and PI. These observations are consistent with the fact that CXCR4 can also promote survival and proliferation of pancreatic carcinoma cells (Koshiba *et al*, 2000; Marchesi *et al*, 2004; Saur *et al*, 2005; Meijer *et al*, 2008). In contrast, CXCR7 EI score was not correlated with PI. *In vitro,* CXCR7 activation induces proliferation in pancreatic, colorectal, lung and mammary carcinoma cells (Miao *et al*, 2007; Meijer *et al*, 2008). Surprisingly, despite the strong *in vitro* effects, CXCR7 did not influence colorectal tumour growth *in vivo*, at least in a mouse xenograft model (Meijer *et al*, 2008), whereas CXCR7 RNAi significantly reduced the tumour growth of lung and mammary carcinomas (Miao *et al*, 2007). These observations suggest that the influence of CXCR7 on tumours growth is not generally applicable to carcinomas and indicate potential inconsistency between *in vitro* and *in vivo* results.

In our study, CXCR4 tumour overexpression was associated with a higher risk of LN metastasis, liver recurrence and was found to be an independent negative prognosis factor for OS but not for DFS. Although the rate of global recurrence was similar between CXCR4^high^ and CXCR4^low^ tumours, occurrence of liver metastasis, was significantly more frequent in CXCR4^high^ than CXCR4^low^; this can explain why CXCR4 expression failed to strongly predict DFS whereas it appears to be a very good predictor for OS. CXCL12, which is produced in a high level in the LN and the liver can initiate and facilitate the homing of pancreatic cancer cells in these tissues (Raman *et al*, 2007). Our results corroborate with a relevant influence of CXCR4 on proliferation and haematogenous dissemination of PA *in vivo* and strengthen murine *in vivo* studies (Koshiba *et al*, 2000; Marchesi *et al*, 2004; Kaifi *et al*, 2005; Saur *et al*, 2005).

Recently, a study of Maeda *et al* investigating CXCR4 expression in a PA patient population did not report a significant difference in the 5-year survival rate between the patients with positive and those with negative CXCR4 tumour expression (Maeda *et al*, 2008). However, the prognostic value analysis of CXCR4 was restricted to a specific CD133-positive subgroup of PA and not to the whole population. Furthermore, Maeda *et al* also included patients with R1 resection; these conditions can explain the survival difference we presently have observed.

In our series, CXCR7 overexpression was not found as an independent prognostic factor for DFS or OS. Many reasons could explain these differences between CXCR4 and CXCR7 in predicting survival despite the fact that they both bind CXCL12. Unlike CXCR4, CXCL12 activation of CXCR7 does not induce calcium mobilisation and cell migration (Burns *et al*, 2006) but rather results in a proliferative effect and increased adhesion properties (Miao *et al*, 2007; Sierro *et al*, 2007; Wang *et al*, 2008). Furthermore, CXCR4 and CXCR7 have different CXCL12 binding domains (Dambly-Chaudiere *et al*, 2007) and distinct roles during development (Sierro *et al*, 2007). Although the CXCL12/CXCR4 and CXCL12/CXCR7 axes are important factors in cancer cell survival, differences exist between their roles in cancer, and whether they function independently or synergistically should be determined.

Interestingly, combining analysis of CXCR4 and CXCR7 coexpression allowed us to identify a subgroup of PA patients (CXCR4^low^/CXCR7^low^) with a remarkably good prognosis.

Our results indicate that CXCR4 and CXCR7 could be attractive targets for PA therapy. Small molecule CXCR4 antagonists are currently being tested in phase I/II clinical trials and could be attractive therapeutic candidates to combine with gemcitabine in future clinical trials.

In conclusion, CXCR4 immunostaining of pancreatic cancer can serve as a prognostic marker for survival after curative surgery. If confirmed in larger prospective series, this assay may assist the clinicians to select those patients who require adjuvant treatment and may represent an attractive target for adjuvant therapy.

## Figures and Tables

**Table 1:** Baseline patient characteristics (*n*=71)

**Variable**	**No. of patients (%)**
Total	71
Median age (range); years	64.5 (39–81)
	
*Gender*	
Male	39 (55)
Female	32 (45)
	
*ECOG PS*	
0	67 (94)
1	4 (6)
	
*Histologic grade*	
Poorly differentiated	15 (21)
Well, moderately differentiated	56 (79)
	
*Tumour stage*	
T_1_-T_2_	10 (14.1)
T_3_-T_4_	61 (85.9)
	
*Lymph node metastasis*	
Yes	48 (68)
None	23 (32)
	
*Adjuvant treatment*	
Radiochemotherapy	30 (42)
Gemcitabine monotherapy	12 (17)
	
*Tumour recurrence*	
Local	11 (15.5)
Distant	33 (46.5)
Local and distant	13 (18.3)

ECOG PS=Eastern Cooperative Oncology Group performance status.

**Table 2:** CXCR4, CXCR7, HIF-1*α* expressions: EI score per area

	**Tumour (TB +IM)**	**LN**
*CXCR4*		
Median	3.5	4.0
Range	0.4–5.4	0–6
Patients assessable	71	37
Positive (EI score >0)	60 (84.5%)	24 (65.8%)
Low/high	32/39	
		
*CXCR7*		
Median	4.0	2.0
Range	0.3–7.7	0–4
Patients assessable	71	37
Positive (EI score >0)	53 (74.6%)	22 (59.5%)
Low/high	29/42	
		
*HIF-1α*		
Median	3.7	2.6
Range	0.1–8.6	0–9
Patients assessable	71	37
Positive (EI score >0)	48 (67.6%)	19 (51.4%)
Low/high	33/38	

HIF-1*α*=hypoxia inducible factor-1*α*; IM=invasive margin; LN=lymph nodes; TB=tumour bulk.

**Table 3:** Correlation between CXCR4 expression and clinicopathologic factors in pancreatic adenocarcinoma

	**CXCR4 expression**		
**Variables**	**Low *n*=32 (%)**	**High *n*=39 (%)**	***P*-value**
Age, mean ±s.d.	61.3±10.8	64.4±10.4	0.71
			
*Gender*			
Male	16 (50)	23 (59.0)	
Female	16 (50)	16 (41.0)	0.48
			
*Tumour size*			
T1–T2	4 (12.5)	6 (15.4)	
T3–T4	28 (87.5)	33 (84.6)	1.00
			
*Tumour differentiation*			
Well/moderate	24 (87.5)	32 (82.1)	
Poor	8 (12.5)	7 (17.9)	0.56
			
*Lymph node*			
N0	16 (50.0)	7 (17.9)	
N+	16 (50.0)	32 (82.1)	0.015
			
*Liver metastasis* a			
Present	9 (28.1)	23 (59.0)	
Absent	23 (71.9)	16 (41.0)	0.016
			
*Vascular embols*			
Present	9 (28.1)	9 (76.9)	
Absent	23 (71.9)	30 (23.1)	0.78
			
*Lymphatic embols*			
Present	14 (43.8)	25 (64.1)	
Absent	18 (56.2)	14 (35.9)	0.09
PI, mean±s.d.	18.5±9.86	26.6±9.39	0.001

PI=proliferative index.

a Recurrence during follow-up.

**Table 4:** Overall survival and disease-free survival: univariate analysis

	**DFS**		**OS**	
**Variables**	**OR (95% CI)**	***P* value**	**OR (95% CI)**	***P* value**
Age	—	0.25	—	0.34
ECOG PS (0 *vs* 1)	—	0.31	—	0.42
Sex	—	0.53	—	0.56
Poorly differentiated tumour	2.17 (1.04–4.61)	0.04	2.32 (1.12–4.81)	0.031
T_1_,T_2_ *vs* T_3_,T_4_	—	0.33	—	0.20
Lymph node metastasis	2.94 (1.44–6.13)	0.003	2.16 (1.11–4.18)	0.02
Adjuvant treatment	—	0.35	—	0.21
CXCR7^T Low^ (*n*=29)^a^	0		0	
CXCR7^T high^ (n=42)	1.51 (0.80–2.87)	0.20	1.81 (0.91–3.60)	0.12
CXCR4^T low^ (*n*=32)^a^	1		1	
CXCR4^T high^ (*n*=39)	2.12 (0.98–3.82)	0.067	5.55 (1.92–12.31)	0.0006
HIF-1*α*^T low^ (*n*=26)^a^	1		1	
HIF-1*α*^T high^ (*n*=35)	1.11 (0.61–2.02)	0.72	1.54 (0.82–2.94)	0.18

DFS=disease-free survival; OS=overall survival; ECOG PS=Eastern Cooperative Oncology Group performance status; CXCR7^T^=CXCR7 expression in the tumour; CXCR4^T^=CXCR4 expression in the tumour; HIF-1*α*=hypoxia-inducible factor-1*α*; HIF-1*α*^T^=HIF-1*α* expression in the tumour.

a The analysis of EI score as a continuous variable did not modify the statistical results.

**Table 5:** Disease free survival and overall survival: multivariate analysis

**Variables**	**Odds ratio**	**95% CI**	***P* value**
*Disease-free survival: Cox regression model*			
Lymph node metastasis			
Absent	1		
Present	3.03	1.49–6.17	0.002
Tumour differentiation			
Well/moderate	1		
Poor	2.40	1.14–5.05	0.021
CXCR4^a^ EI score			
Low	1		
High	1.86	0.99–3.49	0.054
			
*Overall survival: Cox regression model*			
Lymph node metastasis			
Absent	1		
Present	2.88	1.34–6.21	0.007
Tumour differentiation			
Well/moderate	1		
Poor	2.24	0.95–5.49	0.11
CXCR4 EI score^a^			
Low	1		
High	2.54	1.27–5.10	<0.001

CI=Confident interval.

a The analysis of the CXCR4 EI score as a continuous variable did not modify results of the multivariate analysis.
