# High expression of CXCR4 may predict poor survival in resected pancreatic adenocarcinoma

**DOI:** 10.1038/sj.bjc.6605894

**Published:** 2010-09-07

**Authors:** R Maréchal, P Demetter, N Nagy, A Berton, C Decaestecker, M Polus, J Closset, J Devière, I Salmon, J-L Van Laethem

**Correction to:** *British Journal of Cancer* (2009) **100**, 1444–1451; doi:10.1038/sj.bjc.6605020

Upon publication of this paper in 2009, the authors noticed two errors – one in the abstract and one within the Results section.

On page 1444 (Abstract), the seventh sentence should read as follows:

‘In a combining analysis, patients with a CXCR7^high^/CXCR4^high^ tumour had a significantly shorter DFS and OS than patients with a CXCR4^low^/CXCR7^low^ tumour’.

On page 1447 (Results), the first sentence of the section entitled ‘CXCR4 and CXCR7 coexpression and patients outcome’ should read as follows:

‘Interestingly, patients with CXCR4^low^/CXCR7^low^ tumour expression have a significantly prolonged DFS and OS than those patients with a CXCR4^high^/CXCR7^high^ tumour expression (Figure 3C; median DFS: 15.80 months (95% CI: 10.43–21.17) *vs* 7.62 (2.97–12.27), *P*=0.037; median OS: not reached *vs* 9.69 (95% CI: 5.13–14.07), *P*=0.001)’.

